# Editorial: ATF3: a crucial stress-responsive gene of glia and neurons in CNS

**DOI:** 10.3389/fnmol.2024.1484487

**Published:** 2024-10-17

**Authors:** Ronit Heinrich, Ami Aronheim, Yung-Chih Cheng, Ido Perlman

**Affiliations:** ^1^Ruth & Bruce Rappaport Faculty of Medicine, Technion, Haifa, Israel; ^2^Emerging Science and Innovation, Worldwide Research, Pfizer Cambridge, Cambridge, MA, United States

**Keywords:** ATF3, CNS, nerve injury, apoptosis, neuronal regeneration, axonal injury

The central nervous system (CNS) is a highly complex and sensitive structure that responds dynamically to various stressors, including physical injuries (Weil et al., 2008), psychological stress (Pavlovsky et al., 2013), and neuroinflammatory conditions (Förstner et al., 2018). Within this complicated system, transcription factors play pivotal roles in modulating gene expression in response to different stressors, with Activating Transcription Factor 3 (ATF3) emerging as a crucial player in the CNS response to stress (Anderson, 2012). This editorial emphasizes the significance of ATF3 in CNS stress, highlighting recent research findings and their implications for neurological health and disease.

ATF3 is a member of the ATF/cAMP responsive element-binding (CREB) protein family and is widely recognized as a stress-inducible gene (Hai et al., 1999). It is rapidly upregulated in response to various stress signals, including axonal injury (Wong et al., 2018), oxidative stress (Okamoto et al., 2006), ischemia (Kao et al., 2023), metabolic stress (Ku and Cheng, 2020) and mechanical injury (Wong et al., 2018; Lou et al., 2023).

Recently, ATF3 was shown to be induced specifically in neurons of the spinal cord or cortex within 1 day after **s**pinal **c**ord **i**njury (SCI) or ischemic stroke in mice (Kao et al., 2023; Pan et al., 2024). Additionally, ATF3 protein levels in mouse blood significantly increased 1 day after SCI as well as after ischemic stroke (Pan et al., 2024). Most importantly, ATF3 protein levels in human serum were elevated in patients within 24 h after SCI or ischemic stroke (Pan et al., 2024). These results demonstrate the feasibility of using ATF3 level in patients' serum as a potential biomarker for CNS trauma (Pan et al., 2024).

One of the primary contexts in which ATF3 has been studied is neuronal injury (Tsujino et al., 2000). Following axonal damage, ATF3 expression is markedly increased in affected neurons, which is believed to contribute to the cellular response aimed at repair and regeneration (Petrović et al., 2022). For instance, in models of peripheral nerve injury, ATF3 has been shown to promote neurite outgrowth and to enhance the intrinsic growth capacity of neurons (reviewed in Katz et al., 2022). Comparing RNA expression data across species that exhibit different abilities to regenerate their nervous system following traumatic nerve injury reveals that ATF3 is consistently induced in neurons within the first few days after injury. Thus, ATF3 can definitely be considered as an evolutionary conserved regulator of neuronal regeneration (Katz et al., 2022).

ATF3 also modulates the response of the CNS to inflammation and psychological stress. In conditions of neuroinflammation, such as those observed in multiple sclerosis and other neurodegenerative diseases, ATF3 expression is upregulated in glial cells (reviewed in Anderson, 2012), including astrocytes and microglia. This upregulation is associated with the modulation of inflammatory responses (Hai et al., 2018), which can be either protective or detrimental depending upon the context and duration of the stressor. It has been mentioned that targeting microglial ATF3 upregulation to mitigate inflammation would be an interesting and necessary therapeutic avenue across a range of CNS disease (Holland and Ramer). Regarding cell death, ATF3 has been reported to be either pro-apoptotic or anti-apoptotic. For instance, the neuroinflammatory response during Alzheimer disease (AD) is triggered by microglia secreting pro-inflammatory cytokines, including CCL4, which attracts astrocytes to encapsulate plaques. Noteworthy, CCL4 is undetectable in normal brain, however, it is significantly present in AD brain at increased concentrations and is positively correlated with amyloid deposition. As ATF3 was found to bind to the promoter region of the CCL4 gene and to positively regulate its transcription, it was concluded that AD progression is controlled also by ATF3 which regulates the associated neuroinflammation pathways (reviewed in Yang et al., 2023). In another study of a mouse model of Parkinson's diseases, ATF3-knockdown decreased CHOP (C/EBP-homologous protein) and cleaved caspase-3 levels (apoptotic markers), suggesting that ATF3 plays a role in apoptosis induction (Zhao et al., 2016). Additionally, in response to lipotoxic brain microvascular damage, ATF3 was found to mainly govern triglyceride-rich lipoprotein-induced inflammation and TNF signaling in the cerebrovascular system and increase endothelial cell apoptosis (Nyunt et al., 2019). However, in ischemic stroke, ATF3 overexpression attenuated neuronal caspase-dependent apoptosis, microglial activation, and pro-inflammatory cytokine production to alleviate brain injury. ATF3 was also shown to reduce microglia activation and, by doing so, to prevent apoptosis. In addition, ATF3 was shown to prevent neuronal apoptosis by promoting the expression of the anti-apoptotic neuronal survival factor HSP27 and activation of Akt (reviewed in Li et al., 2023).

Recent studies have explored the role of ATF3 in psychological stress response of the CNS. Chronic stress is known to induce changes in brain function and structure, contributing to mental health disorders like depression and anxiety (Green et al., 2008; Pai et al., 2018). ATF3 appears to be involved in the cellular mechanisms underlying these changes, influencing neuroplasticity and stress resilience.

The multifaceted roles of ATF3 in the CNS suggest that it could become a valuable target for therapeutic interventions aimed at mitigating the effects of CNS stress. For example, enhancing ATF3 expression in neurons might promote recovery after traumatic brain injury or stroke (reviewed in Li et al., 2023). Additionally, modulating ATF3 activity in glial cells could help manage chronic neuroinflammation seen in diseases like Alzheimer's (Yang et al., 2023) and Parkinson's (Yoo et al., 2017).

However, the dual nature of ATF3's actions—promoting repair in some contexts (Seijffers et al., 2006) while potentially exacerbating damage in others (Yang et al., 2023)—necessitates a nuanced approach to therapeutic development. Understanding the precise conditions under which ATF3 exerts its beneficial vs. detrimental effects will be crucial for harnessing its potential in clinical settings.

## Conclusion

ATF3 is a pivotal transcription factor in the CNS response to all forms of stress, playing significant roles in neuronal injury repair, neuroinflammation, recovery from metabolic stress, and psychological stress adaptation. Ongoing research continues to unravel the complexities of ATF3 functions, indicating its actions to promote functional tissues repair via two opposing pathways. In some cases, it acts to promote regeneration of damaged tissue while in others it may activate apoptosis of badly damaged cells to prevent spread of damage to non-affected cells in the same tissue. The dual nature of ATF3 actions is highly context dependent. Its effects vary based on the type of stress, the specific tissue or cell type, and the interplay with other signaling pathways. Thus, ATF3 offers promising avenues for therapeutic interventions aimed at enhancing CNS resilience and recovery. As we deepen our understanding of ATF3 roles and mechanisms, we move closer to developing targeted strategies that can attenuate the adverse effects of different types of stress on CNS function and improve outcomes for individuals suffering from neurological disorders.
